# Synthesis, X-ray diffraction study, analysis of inter­molecular inter­actions and mol­ecular docking of ethyl 1-(3-tosyl­quinolin-4-yl)piperidine-4-carboxyl­ate

**DOI:** 10.1107/S2056989022007691

**Published:** 2022-08-09

**Authors:** Yevhenii Vaksler, Halyna V. Hryhoriv, Sergiy M. Kovalenko, Lina O. Perekhoda, Victoriya A. Georgiyants

**Affiliations:** a SSI Institute for Single Crystals, National Academy of Sciences of Ukraine, 60 Nauky Ave, Kharkov 61001, Ukraine; b The National University of Pharmacy, 53 Pushkinska St., Kharkiv 61002, Ukraine; c V. N. Karazin Kharkiv National University, 4 Svobody Sq., Kharkiv 61077, Ukraine; Indian Institute of Science Education and Research Bhopal, India

**Keywords:** ethyl 1-(3-tosyl­quinolin-4-yl)piperidine-4-carboxyl­ate, anti­bacterial properties, mol­ecular and crystal structure, Hirshfeld surface analysis, pairwise inter­action energy, mol­ecular docking

## Abstract

The title compound, C_24_H_26_N_2_O_4_S, can be obtained *via* two synthetic routes. According to our investigations, the most suitable way is by the reaction of ethyl 2-bromo­acetate with sodium tosyl­sulfinate in dry DMF. It was crystallized from methanol into the monoclinic *P*2_1_/*n* space group with a single mol­ecule in the asymmetric unit. Hirshfeld surface analysis was performed to define the hydrogen bonds and analysis of the two-dimensional fingerprint plots was used to distinguish the different types of inter­actions. Two very weak non-classical C—H⋯O hydrogen bonds were found and the contributions of short contacts to the Hirshfeld surface were determined. Mol­ecules form an isotropic network of inter­molecular inter­actions according to an analysis of the pairwise inter­action energies. A mol­ecular docking study evaluated the inter­actions in the title compound with the active centers of macromolecules of bacterial targets (*Staphylococcus aureus* DNA Gyrase PDB ID: 2XCR, *Mycobacterium tuberculosis topoisomerase* II PDB ID: 5BTL, *Streptococcus pneumoniae topoisomerase* IV PDB ID: 4KPF) and revealed high affinity towards them that exceeded the reference anti­biotics of the fluoro­quinolone group.

## Abstract 2

An easy synthetic route towards ethyl 1-(3-tosyl­quinolin-4-yl)piperidine-4-carboxyl­ate was found. Its mol­ecular and crystal structures are described as well and the biological activity is also predicted using mol­ecular docking studies.

## 1. Chemical context

Quinolone-based compounds have become strikingly conspicuous in recent years. Generally, quinolone derivatives can possess anti­bacterial, anti­parasitic and anti­viral (including malaria, hepatitis, HIV, herpes), anti­cancer and immunosuppressant activities. They can be used in the treatment of obesity, diabetes and neurodegenerative diseases (Horta *et al.*, 2017 ▸). Thus, in this work, we decided to broaden the scope of the quinolone scaffolds utilized in our previous works (Bylov *et al.*, 1999 ▸; Silin *et al.*, 2004 ▸; Savchenko *et al.*, 2007 ▸; Hryhoriv *et al.*, 2021 ▸) toward a promising new class of aryl­sulfonyl­quinolin derivatives, namely ethyl 1-(3-tosyl­quinolin-4-yl)piperidine-4-carboxyl­ate.

Effective synthetic approaches toward these compounds are versatile. The most notable among them are green chemistry methods and microwave-assisted synthesis (Dhiman *et al.*, 2019 ▸; Atechian *et al.* 2007 ▸). However, to date, very few data are available for aryl­sulfonyl­quinolins. Kang *et al.* (2016 ▸) described a straightforward and mild one-pot method to synthesize 3-(phenyl­sulfon­yl)-2,3-di­hydro-4(1*H*)quinolino­nes *via* a Cu-catalyzed aza-Michael addition/base-mediated cycliz­ation reaction. Other researchers (Ivachtchenko *et al.* 2012*a* ▸,*b* ▸) have reported new 3-(phenyl­sulfon­yl)quinoline derivatives as serotonin 5-HT receptor antagonists, performed mol­ecular docking studies, and proposed them for preventing and treating central nervous system (CNS) diseases such as psychiatric disorders, schizophrenia, anxiety disorders, and obesity. The preparation method for 3-methane­sulfonyl­quinolines such as GABA-B enhancers was patented by Malherbe *et al.* (2006 ▸). *In vivo* investigations of 4-amino-3-aryl­sulfoquinolin derivatives as metabotropic glutamate 5(mGlu) receptor negative allosteric modulators have shown efficacy for treating anxiety and depression (Galambos *et al.*, 2017 ▸). 
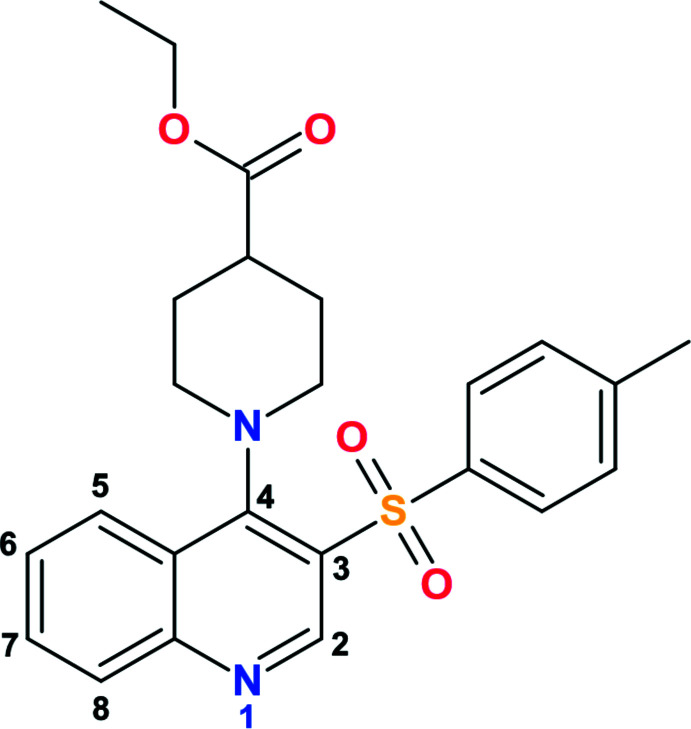


In the present paper, we study an optimal synthetic route for ethyl 1-(3-tosyl­quinolin-4-yl)piperidine-4-carboxyl­ate and report its mol­ecular and crystal structures as well as potential biological properties.

## 2. Structural commentary

**Figure 1:**
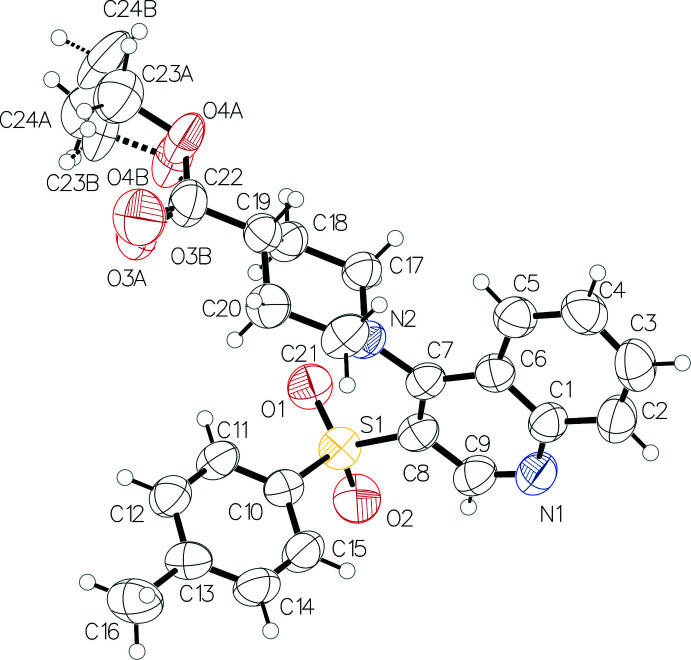
Mol­ecular structure of the title compound. Displacement ellipsoids are shown at the 50% probability level.

The asymmetric unit contains one mol­ecule of ethyl 1-(3-tosyl­quinolin-4-yl)piperidine-4-carboxyl­ate (Fig. 1 ▸). The presence of two bulky substituents in vicinal positions at the pyridine ring results in a rotation of the piperidine ring with respect to the bicyclic fragment [the dihedral angle between their mean planes is 76.83 (13)°]. The piperidine ring adopts a chair conformation with puckering parameters (Zefirov *et al.*, 1990 ▸) *S* = 1.16 (1), *Θ* = 0.6 (1)°, *Ψ* = 66.2 (12)°. The atoms N2 and C19 deviate from the mean plane of the other ring atoms by −0.640 (2) and 0.675 (3) Å, respectively. The atom N2 has a pyramidal configuration with a bond-angle sum of 345.4°. The ethyl ester group is located in an equatorial position with respect to the piperidine ring [the C17—C18—C19—C22 torsion angle is 179.8 (2)°]. It is disordered over the two positions (*A* and *B*) due to rotation around the C19—C22 bond with an occupancy ratio of 0.562 (12):0.438 (12). The ethyl group is almost orthogonal to the carb­oxy­lic fragment in conformer *A* and is located in the inter­mediate position between +*ac* and *ap* in conformer *B* [the C22—O4—C23—C24 torsion angle is −98.1 (14) and 150 (2)° in conformers *A* and *B*, respectively]. The tolyl substituent is located in a -*sc* position relative to the endocyclic C7—C8 bond [C7—C8—S1—C10 = −71.5 (3)°] and rotated about the C8—S1 bond [C8—S1—C10—C11 = 124.9 (2)°].

## 3. Supra­molecular features

**Figure 2:**
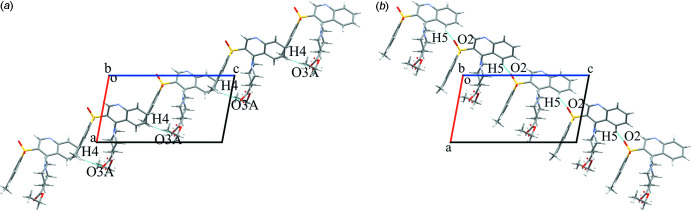
Crystal packing of the chains built with the hydrogen bonds C4—H4⋯O3*A* (*a*) and C5—H5⋯O2 (*b*) in cyan. Projection in the [010] direction.

Regarding the van der Waals radii proposed in Bondi (1964 ▸) for all atoms except for the hydrogens (Rowland & Taylor, 1996 ▸), the analysis of inter­molecular inter­actions revealed two very weak non-classical hydrogen bonds, C4—H4⋯O3*A* and C5—H5⋯O2 (Table 1 ▸). The first is formed by an oxygen atom of the carb­oxy­lic group and a hydrogen atom of the benzene ring (Fig. 2 ▸ *a*). An oxygen atom of the sulfonyl group is involved in the second hydrogen bond, similarly with a hydrogen atom of the benzene ring (Fig. 2 ▸ *b*). Connected with the initial mol­ecule by the symmetry operations *x* − 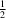 , −*y* + 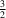 , *z* + 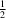 and *x* + 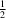 , −*y* + 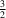 , *z* + 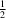 , these hydrogen bonds are affected by both twofold screw axes <010> and glide plane family {010}. On their own, these hydrogen bonds form the chains in the [10 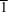 ] and [101] directions, respectively.

## 4. Hirshfeld surface analysis

**Figure 3:**
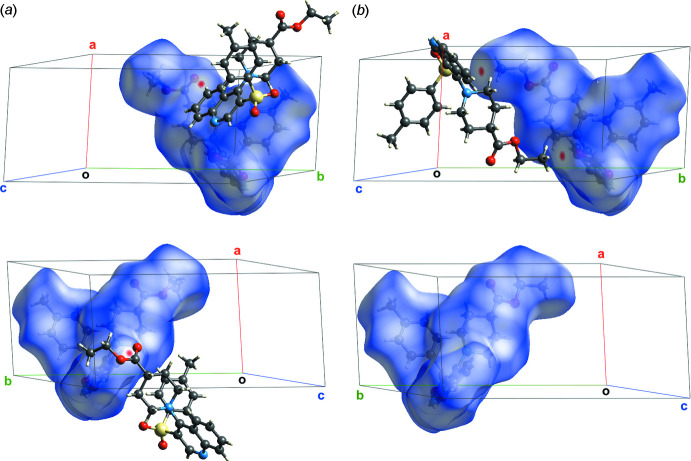
Distribution of the value *d*_norm_ onto the Hirshfeld surfaces of the conformers *A* (*a*) and *B* (*b*).

The complementation of the Hirshfeld surface, *i.e*. the surface splitting the regions of crystal into mol­ecular domains within the ratio of promolecular to procrystal electronic density, with geometric parameters, especially the normalized contact distance (*d*_norm_), implemented in *CrystalExplorer17* (Spackman *et al.*, 2021 ▸) allowed us to distinguish the inter­molecular inter­actions in a more thorough way. The standard ‘high’ surface resolution was used. Two regions with *d*_norm_ significantly lower than the van der Waals contact length (in red) emerge on the surface (Fig. 3 ▸ *a*). Both of them concern the C4—H4⋯O3*A* hydrogen bond and show it to be the sole directed inter­action in the crystal. The chains built up by these hydrogen bonds are parallel to the [ 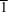 01] direction. However, they cannot be considered as a structural motif because the aforementioned hydrogen bonds are very weak, exist solely for conformer *A* and one of them was not revealed for conformer *B*. At the same time, the short contact C24*B*⋯O1 appears just for conformer *B* (Fig. 3 ▸ *b*). Differences in the distribution of *d*_norm_ for the two conformers and so the short contacts and hydrogen bonds can be easily be seen from the two projections (top and bottom) shown in Fig. 3 ▸.

**Figure 4:**
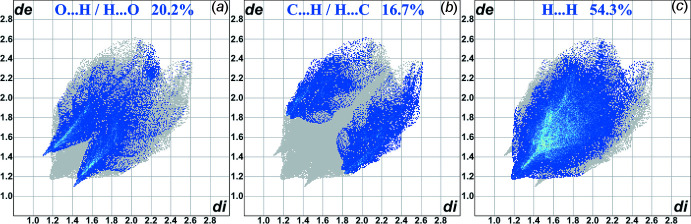
Contributions of the O⋯H/H⋯O (*a*), C⋯H/H⋯C (*b*) and H⋯H (*c*) contacts to the fingerprint plots built using the Hirsfeld surfaces of conformer *A*.

In addition to the Hirshfeld surface analysis, the 2D fingerprint plots were computed for ethyl 1-(3-tosyl­quinolin-4-yl)piperidine-4-carboxyl­ate. The contributions of the three types of inter­molecular contacts get to areas with the values of the inter­nal and external distances (*d*_i_ and *d*_e_) below the van der Waals radii of the corresponding atoms (Fig. 4 ▸). These contributions belong to the short O⋯H, C⋯H and H⋯H contacts. They are 20.2% and 19.9% of the Hirshfeld surface area for H⋯O/O⋯H for the disorder components *A* and *B*, respectively, 16.7% and 17.7%, respectively, for C⋯H/H⋯C and 54.3% for H⋯H. The differences for the disordered positions *A* and *B* can be explained by the rearrangement of the inter­actions network described above.

## 5. Analysis of the pairwise inter­action energies

**Figure 5:**
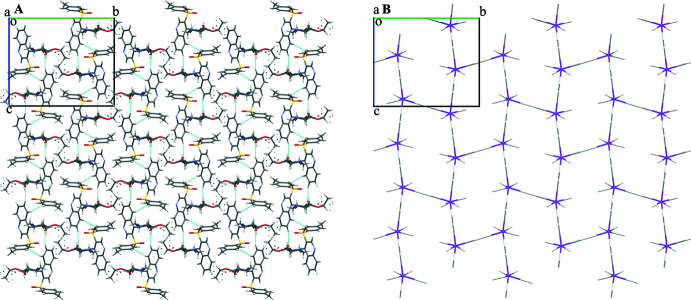
Crystal packing of the mol­ecules (*a*) and energy vector diagrams (*b*). Projection in the [100] direction.

The strength of the non-classical C—H⋯O hydrogen bonds is often underestimated, as mentioned in Sutor (1962 ▸) and Desiraju (1996 ▸, 2005 ▸). Thus, to extend the knowledge of the supra­molecular structure of the title compound and to prove the small contribution of these inter­actions to the structure, analysis of the pairwise inter­action energies was performed as proposed by Konovalova *et al.* (2010 ▸) and Shishkin *et al.* (2012 ▸). The procedure was implied in a very similar way to the one described in detail in Vaksler *et al.* (2021 ▸). The single mol­ecule was considered as a building unit. The inter­actions in the mol­ecular pairs containing the aforementioned hydrogen bonds are −7.4 and −10.5 kcal mol^−1^ for conformer *A* and −3.2 and −11.7 kcal mol^−1^ for conformer *B* (data given for the C24*B*⋯O1 short contact and the C5—H5⋯O2 hydrogen bond). These values are comparable to those for the non-directed inter­actions in other pairs of neighboring mol­ecules. In addition to this, the inter­action energy decomposition was performed using an ‘accurate’ energy model in *CrystalExplorer17* for the mol­ecular pairs with C—H⋯O hydrogen bonds. It showed that the sum of electrostatic and polarization components is rather low in comparison with the dispersion and repulsion terms (−1.0 *versus* −7.5 and 2.6 kcal mol^−1^ for the C4—H4⋯O3*A* hydrogen bond, −4.6 versus −9.7 and 4.2 kcal mol^−1^ for C5—H5⋯O2) implying minimal contributions of hydrogen bonds in general bonding. Despite the apparent layering (Fig. 5 ▸ *a*) parallel to the (010) plane, the energetic structure of the title compound can be considered isotropic, which can easily be seen from the energy vector diagrams (Fig. 5 ▸ *b*). The total energy of inter­action between a basic mol­ecule and its first coordination sphere is −95.8 and −95.5 kcal mol^−1^ for conformers *A* and *B*, respectively.

## 6. Database survey

A search of the Cambridge Structural Database [Version 5.42, update of November 2020; Groom *et al.*, 2016 ▸] shows no similarities between the title compound and 4-(piperidin-1-yl)-3-sulfone-quinoline derivatives.

## 7. Mol­ecular docking

A mol­ecular docking study was performed in order to estimate the application efficiency of ethyl 1-(3-tosyl­quinolin-4-yl)piperidine-4-carboxyl­ate in terms of medicinal chemistry as anti­microbials. For receptor-oriented flexible docking, the *Autodock 4.2* software package (Morris *et al.*, 2009 ▸) was used. Ligands were prepared using the *MGL Tools 1.5.6* (Sanner, 1999 ▸) and optimized within the *Avogadro* (Hanwell *et al.*, 2012 ▸) (United Force Field with the steepest descent algorithm). The biotargets were chosen on the basis of structural similarity between the title compound and known anti­bacterial agents from the group of fluoro­quinolones. The active centers of macromolecules of bacterial targets [*Staphylococcus aureus* DNA Gyrase PDB ID: 2XCR (Bax *et al.*, 2010 ▸); *Mycobacterium tuberculosis topoisomerase II* PDB ID: 5BTL (Blower *et al.*, 2016 ▸); *Streptococcus pneumoniae topoisomerase IV* PDB ID: 4KPF (Laponogov *et al.*, 2016 ▸)] from the Protein Data Bank (PDB) were used for docking.

The receptor maps were made with *MGL Tools* and *AutoGrid* (Sanner, 1999 ▸). The docking parameters were defined closely to the ones mentioned in Syniugin *et al.* (2016 ▸; see supporting information). These parameters were chosen to bring the formation of a complex between the tested mol­ecule and the receptor as close as possible to the conditions that exist in biological systems.

Inhibitory activity against bacterial targets can be realized by the formation of their complexes with ligands [as ethyl 1-(3-tosyl­quinolin-4-yl)piperidine-4-carboxyl­ate]. In turn, the stability of complexes can be estimated from the strength of the inter­molecular inter­actions. The scoring function indicating the enthalpy contribution to the value of the free binding energy (affinity DG), the values of the free binding energy and binding constants [*E*_Doc_ (kcal mol^−1^) and *K_i_* (μM)] are represented for the most profitable conformation positions (Table 2 ▸). All the parameters show that the title compound is superior to the reference medicines of the same type.

## 8. Synthesis and crystallization

The starting compounds were obtained from commercial sources and were used without further purification.

Two ways were proposed for the synthesis of ethyl 1-(3-tosyl­quinolin-4-yl)piperidine-4-carboxyl­ate: the classical one and an alternative one with a lower number of steps and higher yield of the final product:

Classical synthesis. In the first stage, the addition of methyl propiolate **2** to aniline **1** produces labile *cis–trans* mixtures of enamine **3**. Thermal cyclization of enamine provides a synthesis of 4(1*H*)-quinolone **4** (Gray *et al.*, 1951 ▸). 
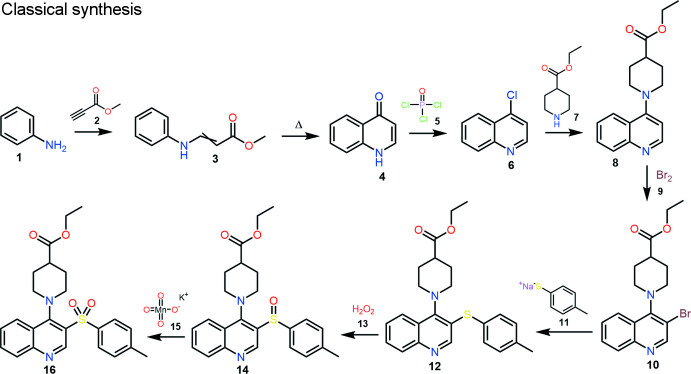


Conversion of 4-hy­droxy­quinoline **4** to 4-chloro­quinoline **6** can be carried out by a known halogenation method with POCI_3_ **5**, or other suitable reagents (*e.g.* SOCI_2_, PCI_5_, POBr_3_, PBr_3_). The obtained 4-chloro­quinoline **6** can be converted to 4-amino­quinoline derivative **8** by an aromatic nucleophilic substitution reaction with secondary amine **7**. Standard bromination of quinoline **8** gives the product **10**. 4-Amino-3-bromo­quinoline **10** can be substituted by the sodium salt of thio­phenol **11** to provide compound **12**. Oxidation of 4-amino-3-aryl­sulfanyl­quinoline **12** can be accomplished by known methods, preferably in a suitable acid (*e.g.* acetic acid) at 273–278 K with potassium permanganate **15** to give 4-amino-3-aryl­sulfinyl­quinolines **14** or with aqueous hydrogen peroxide **13** in a suitable acid (*e.g.* acetic acid or tri­fluoro­acetic acid). To obtain the title compound **16**, further oxidation of compound **14** is required. The reaction can be carried out by known methods, preferably in a suitable acid (*e.g.* acetic acid) at 273–278 K with potassium permanganate **15** (Keserü *et al.*, 2007 ▸). The yield of the title compound is 46.0%.

Alternative synthesis*.* Ethyl 2-tosyl­acetate **19** is obtained by the reaction of ethyl 2-bromo­acetate **18** with sodium tosyl­sulfinate **17** in dry DMF. Compound **21** can be obtained by the condensation reaction of compound **19** with *N*,*N*-di­methyl­formamide di­methyl­acetal **20** without using a solvent or in a minimum amount of dioxane. Compound **21**, upon reaction with aniline **22** in iso­propanol/AcOH medium, produces an *E*/*Z* isomer mixture of enamine **23**, which is converted to 3-tosyl­quinoline-4-(1*H*)-one **24** by thermal cyclization in diphenyl ether. Chlorination of compound **24** is carried out according to a known method with phospho­rus oxychloride **5**. The final product **16** is obtained by the reaction of aromatic nucleophilic substitution of 4-chloro-3-tosyl­quinoline **25** with ethyl piperidine-4-carboxyl­ate **7** in a dry DMF medium using a base (tri­ethyl­amine, DBU), or excess of secondary amine (Keserü *et al.*, 2007 ▸). The yield of the title compound is 73.6%. Recrystallization by slow evaporation of a solution in aceto­nitrile produced block-like colorless crystals suitable for X-ray diffraction analysis. The advantages of this synthesis make it seem preferable to the common one. 
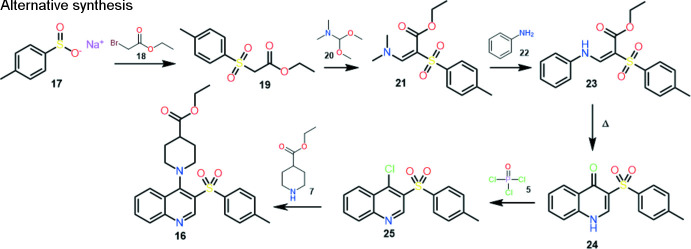


## 9. NMR characterization

The NMR spectra were recorded on a Varian MR-400 spectrometer with standard pulse sequences operating at 400 MHz for ^1^H NMR, 101 MHz for ^13^C NMR. For the NMR spectra, DMSO-*d*_6_ was used as a solvent. Chemical shift values are referenced to residual protons (δ 2.49 ppm) and carbons (δ 39.6 ppm) of the solvent as an inter­nal standard. LC/MS spectra were recorded on a ELSD Alltech 3300 liquid chromatograph equipped with a UV detector (λ_max_ 254 nm), API-150EX mass-spectrometer using a Zorbax SB-C18 column, Phenomenex (100 × 4 mm) Rapid Resolution HT Cartridge 4.6 × 30mm, 1.8-Micron. Elution started with an 0.1 *M* solution of HCOOH in water and ended with an 0.1 *M* solution of HCOOH in aceto­nitrile using a linear gradient at a flow rate of 0.15 ml min^−1^ and an analysis cycle time of 25 min.

Characteristics of the title mol­ecule:

^1^H NMR (400 MHz, DMSO-*d*_6_) δ 9.37 (*s*, 1H), 8.28 (*d*, *J* = 8.6 Hz, 1H), 8.17 (*d*, *J* = 8.4 Hz, 1H), 7.93 (*t*, *J* = 7.7 Hz, 1H), 7.72 (*t*, *J* = 8.0 Hz, 3H), 7.39 (*d*, *J* = 8.0 Hz, 2H), 4.10 (*q*, *J* = 7.1 Hz, 2H), 3.37 (*d*, *J* = 19.2 Hz, 1H), 3.31–3.27 (*m*, 1H), 2.92 (*d*, J = 11.2 Hz, 2H), 2.55 (*d*, *J* = 9.2 Hz, 1H), 2.38 (*s*, 3H), 1.64 (*dd*, *J* = 13.2, 3.8 Hz, 2H), 1.41–1.36 (*m*, 1H), 1.35 (*s*, 1H), 1.22 (*t*, *J* = 7.1 Hz, 3H).

^13^C NMR (101 MHz, DMSO-*d*_6_) δ 174.17, 158.14, 151.56, 149.44, 143.64, 139.23, 132.37, 130.31, 129.66, 127.43, 126.19, 125.86, 59.85, 50.56, 27.26, 21.02, 14.11.

## 10. Refinement

Crystal data, data collection and structure refinement details are summarized in Table 3 ▸. All hydrogen atoms were positioned geometrically (C–H= 0.93–0.97 Å) and refined using a riding model with *U*_iso_(H) = *nU*_eq_ of the carrier atom (*n* = 1.5 for methyl groups and *n* = 1.2 for other hydrogen atoms). During the refinement the distances between the atoms of the disordered part were restrained to the following values: 1.497 Å for the bond C19—C22, 1.196 Å for O3—C22, 1.336 Å for O4—C22, 1.452 Å for O4—C23 and 1.513 Å for C23—C24 according to the mean values in Dunitz & Bürgi (1994 ▸). The estimated standard deviation was set at 0.005 Å for all the bonds.

## Supplementary Material

Crystal structure: contains datablock(s) I. DOI: 10.1107/S2056989022007691/dx2047sup1.cif

Structure factors: contains datablock(s) I. DOI: 10.1107/S2056989022007691/dx2047Isup2.hkl

The parameters used for molecular docking. DOI: 10.1107/S2056989022007691/dx2047sup3.pdf

Supporting information file. DOI: 10.1107/S2056989022007691/dx2047Isup4.cml

CCDC reference: 2193735

Additional supporting information:  crystallographic information; 3D view; checkCIF report

## Figures and Tables

**Table 1:** Hydrogen-bond geometry (Å, °)

*D*—H⋯*A*	*D*—H	H⋯*A*	*D*⋯*A*	*D*—H⋯*A*
C4—H4⋯O3*A* ^i^	0.93	2.52	3.421 (16)	163
C5—H5⋯O2^ii^	0.93	2.58	3.415 (4)	149

Symmetry codes: (i) 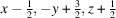 ; (ii) 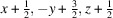 .

**Table 2:** The values of affinity DG, free binding energy, and binding coefficients for the best conformational positions of the title compound in combination with biotargets (PDB ID: 2XCR, 5BTL, 4KPF). Values are also given for reference compounds

Mol­ecule	Affinity DG (kcal mol^−1^)	*E*_Doc_ (kcal mol^−1^)	*K_i_* (υ*M*)
**PDB ID: 2XCR**			
**Title compound**	−7.5	−5.62	76.15
Ciprofloxacin	−7.2	−5.10	183.79
Norfloxacin	−7.2	−4.30	708.28
**PDB ID: 5BTL**			
**Title compound**	−8.2	−5.64	73.02
Ciprofloxacin	−7.5	−5.51	91.69
Norfloxacin	−7.8	−5.25	142.92
**PDB ID: 4KPF**			
**Title compound**	−8.1	−6.13	31.90
Ciprofloxacin	−7.4	−5.38	113.52
Norfloxacin	−7.4	−4.78	315.73

**Table 3:** Experimental details

Crystal data	
Chemical formula	C_24_H_26_N_2_O_4_S
*M*_r_	438.53
Crystal system, space group	Monoclinic, *P*2_1_/*n*
Temperature (K)	298
*a*, *b*, *c* (Å)	8.2608 (3), 17.9433 (7), 15.2470 (7)
β (°)	100.626 (4)
*V* (Å^3^)	2221.25 (16)
*Z*	4
Radiation type	Mo *K*α
μ (mm^−1^)	0.18
Crystal size (mm)	0.3 × 0.2 × 0.1

Data collection	
Diffractometer	Xcalibur, Sapphire3
Absorption correction	Multi-scan (*CrysAlis PRO*; Rigaku OD, 2018 ▸)
*T*_min_, *T*_max_	0.400, 1.000
No. of measured, independent and observed [*I* > 2σ(*I*)] reflections	22680, 6477, 3049
*R*_int_	0.104
(sin θ/λ)_max_ (Å^−1^)	0.703

Refinement	
*R*[*F* ^2^ > 2σ(*F* ^2^)], *wR*(*F* ^2^), *S*	0.077, 0.253, 1.04
No. of reflections	6477
No. of parameters	321
No. of restraints	9
H-atom treatment	H-atom parameters constrained
Δρ_max_, Δρ_min_ (e Å^−3^)	0.33, −0.52

Computer programs: *CrysAlis PRO* (Rigaku OD, 2018 ▸), *SHELXT2014/5* (Sheldrick, 2015*a* ▸), *SHELXL2017/1* (Sheldrick, 2015*b* ▸) and *OLEX2* (Dolomanov *et al.*, 2009 ▸).

## supplementary crystallographic information

### Crystal data

**Table:**

C_24_H_26_N_2_O_4_S	*F*(000) = 928
*M**_r_* = 438.53	*D*_x_ = 1.311 Mg m^−^^3^
Monoclinic, *P*2_1_/*n*	Mo *K*α radiation, λ = 0.71073 Å
*a* = 8.2608 (3) Å	Cell parameters from 2249 reflections
*b* = 17.9433 (7) Å	θ = 3.5–22.9°
*c* = 15.2470 (7) Å	µ = 0.18 mm^−^^1^
β = 100.626 (4)°	*T* = 298 K
*V* = 2221.25 (16) Å^3^	Block, colourless
*Z* = 4	0.3 × 0.2 × 0.1 mm

### Data collection

**Table:**

Xcalibur, Sapphire3 diffractometer	6477 independent reflections
Radiation source: fine-focus sealed X-ray tube, Enhance (Mo) X-ray Source	3049 reflections with *I* > 2σ(*I*)
Graphite monochromator	*R*_int_ = 0.104
Detector resolution: 16.1827 pixels mm^-1^	θ_max_ = 30.0°, θ_min_ = 3.0°
ω scans	*h* = −11→11
Absorption correction: multi-scan (CrysAlisPro; Rigaku OD, 2018)	*k* = −25→24
*T*_min_ = 0.400, *T*_max_ = 1.000	*l* = −21→20
22680 measured reflections	

### Refinement

**Table:**

Refinement on *F*^2^	Hydrogen site location: inferred from neighbouring sites
Least-squares matrix: full	H-atom parameters constrained
*R*[*F*^2^ > 2σ(*F*^2^)] = 0.077	*w* = 1/[σ^2^(*F*_o_^2^) + (0.106*P*)^2^] where *P* = (*F*_o_^2^ + 2*F*_c_^2^)/3
*wR*(*F*^2^) = 0.253	(Δ/σ)_max_ < 0.001
*S* = 1.04	Δρ_max_ = 0.33 e Å^−^^3^
6477 reflections	Δρ_min_ = −0.52 e Å^−^^3^
321 parameters	Extinction correction: SHELXL-2017/1 (Sheldrick 2015b), Fc^*^=kFc[1+0.001xFc^2^λ^3^/sin(2θ)]^-1/4^
9 restraints	Extinction coefficient: 0.0057 (16)
Primary atom site location: structure-invariant direct methods	

### Special details

**Table:**

Geometry. All esds (except the esd in the dihedral angle between two l.s. planes) are estimated using the full covariance matrix. The cell esds are taken into account individually in the estimation of esds in distances, angles and torsion angles; correlations between esds in cell parameters are only used when they are defined by crystal symmetry. An approximate (isotropic) treatment of cell esds is used for estimating esds involving l.s. planes.

### Fractional atomic coordinates and isotropic or equivalent isotropic displacement parameters (Å^2^)

**Table:**

	*x*	*y*	*z*	*U*_iso_*/*U*_eq_	Occ. (<1)
S1	0.12710 (8)	0.79643 (4)	0.44018 (5)	0.0610 (3)	
O1	0.1623 (3)	0.71849 (11)	0.44202 (15)	0.0728 (6)	
O2	−0.0150 (2)	0.82165 (14)	0.37816 (14)	0.0790 (6)	
N1	−0.0721 (3)	0.90921 (14)	0.61830 (19)	0.0700 (7)	
N2	0.3448 (3)	0.76877 (12)	0.62148 (16)	0.0557 (6)	
C1	0.0279 (4)	0.89220 (16)	0.6975 (2)	0.0608 (7)	
C2	−0.0099 (4)	0.92616 (19)	0.7745 (2)	0.0765 (9)	
H2	−0.095862	0.960273	0.769311	0.092*	
C3	0.0792 (5)	0.9093 (2)	0.8568 (3)	0.0852 (10)	
H3	0.055013	0.932402	0.907452	0.102*	
C4	0.2055 (5)	0.8577 (2)	0.8645 (2)	0.0811 (10)	
H4	0.262181	0.844427	0.920871	0.097*	
C5	0.2481 (4)	0.82599 (18)	0.7907 (2)	0.0690 (8)	
H5	0.335630	0.792592	0.797560	0.083*	
C6	0.1626 (3)	0.84274 (15)	0.70451 (19)	0.0571 (6)	
C7	0.2030 (3)	0.81265 (14)	0.62430 (19)	0.0529 (6)	
C8	0.0973 (3)	0.82935 (15)	0.54556 (19)	0.0571 (7)	
C9	−0.0392 (3)	0.87693 (18)	0.5469 (2)	0.0669 (8)	
H9	−0.110214	0.885782	0.493097	0.080*	
C10	0.2968 (3)	0.84632 (15)	0.41666 (17)	0.0549 (6)	
C11	0.4327 (3)	0.80964 (17)	0.3971 (2)	0.0649 (8)	
H11	0.438815	0.757924	0.399994	0.078*	
C12	0.5601 (4)	0.85057 (18)	0.3730 (2)	0.0699 (8)	
H12	0.651240	0.825780	0.359412	0.084*	
C13	0.5542 (4)	0.92746 (17)	0.3688 (2)	0.0676 (8)	
C14	0.4163 (4)	0.96262 (17)	0.3886 (2)	0.0755 (9)	
H14	0.410682	1.014372	0.386521	0.091*	
C15	0.2875 (4)	0.92320 (17)	0.4111 (2)	0.0708 (8)	
H15	0.194724	0.947991	0.422600	0.085*	
C16	0.6935 (4)	0.9711 (2)	0.3433 (3)	0.0968 (12)	
H16A	0.758596	0.992709	0.395828	0.145*	
H16B	0.650002	1.009876	0.302399	0.145*	
H16C	0.761151	0.938456	0.315502	0.145*	
C17	0.3276 (3)	0.68933 (15)	0.6411 (2)	0.0591 (7)	
H17A	0.221619	0.671292	0.610173	0.071*	
H17B	0.332639	0.682536	0.704646	0.071*	
C18	0.4648 (3)	0.64532 (16)	0.6112 (2)	0.0629 (7)	
H18A	0.452600	0.648235	0.546801	0.076*	
H18B	0.456382	0.593353	0.627312	0.076*	
C19	0.6334 (3)	0.67519 (16)	0.65413 (19)	0.0610 (7)	
H19	0.643943	0.669921	0.718917	0.073*	
C20	0.6433 (4)	0.75735 (18)	0.6337 (2)	0.0705 (8)	
H20A	0.636820	0.764020	0.570004	0.085*	
H20B	0.748421	0.776840	0.663831	0.085*	
C21	0.5054 (4)	0.80045 (17)	0.6634 (3)	0.0726 (9)	
H21A	0.517450	0.797924	0.727861	0.087*	
H21B	0.510842	0.852386	0.646572	0.087*	
C22	0.7719 (4)	0.63318 (19)	0.6266 (2)	0.0780 (10)	
O3A	0.8692 (12)	0.6593 (9)	0.5867 (10)	0.110 (4)	0.562 (12)
O4A	0.7720 (9)	0.5613 (2)	0.6503 (6)	0.083 (2)	0.562 (12)
C23A	0.8975 (13)	0.5082 (7)	0.6336 (6)	0.102 (4)	0.562 (12)
H23A	0.925138	0.473765	0.683210	0.123*	0.562 (12)
H23B	0.996805	0.534136	0.625668	0.123*	0.562 (12)
C24A	0.8233 (12)	0.4672 (7)	0.5499 (7)	0.112 (3)	0.562 (12)
H24A	0.812699	0.500471	0.499914	0.169*	0.562 (12)
H24B	0.716630	0.448644	0.555390	0.169*	0.562 (12)
H24C	0.893277	0.426205	0.541008	0.169*	0.562 (12)
O3B	0.9009 (14)	0.6554 (14)	0.6125 (15)	0.132 (7)	0.438 (12)
O4B	0.7400 (14)	0.5610 (4)	0.6108 (11)	0.135 (6)	0.438 (12)
C23B	0.888 (2)	0.5271 (7)	0.5881 (13)	0.122 (6)	0.438 (12)
H23C	0.986565	0.552319	0.618191	0.147*	0.438 (12)
H23D	0.884938	0.528434	0.524241	0.147*	0.438 (12)
C24B	0.8811 (16)	0.4482 (5)	0.6209 (14)	0.124 (6)	0.438 (12)
H24D	0.782857	0.424517	0.589912	0.187*	0.438 (12)
H24E	0.880177	0.448590	0.683798	0.187*	0.438 (12)
H24F	0.975808	0.421342	0.609985	0.187*	0.438 (12)

### Atomic displacement parameters (Å^2^)

**Table:**

	*U*^11^	*U*^22^	*U*^33^	*U*^12^	*U*^13^	*U*^23^
S1	0.0576 (4)	0.0605 (5)	0.0624 (5)	−0.0034 (3)	0.0045 (3)	−0.0067 (3)
O1	0.0859 (15)	0.0524 (12)	0.0821 (16)	−0.0119 (10)	0.0206 (12)	−0.0138 (10)
O2	0.0629 (13)	0.1024 (18)	0.0651 (13)	0.0003 (11)	−0.0052 (10)	−0.0052 (12)
N1	0.0643 (15)	0.0667 (16)	0.0800 (18)	0.0106 (12)	0.0159 (13)	−0.0014 (13)
N2	0.0525 (13)	0.0452 (12)	0.0690 (15)	−0.0003 (9)	0.0105 (11)	−0.0004 (10)
C1	0.0627 (16)	0.0499 (15)	0.0722 (19)	0.0008 (12)	0.0184 (14)	−0.0022 (13)
C2	0.079 (2)	0.073 (2)	0.083 (2)	0.0051 (16)	0.0285 (18)	−0.0110 (17)
C3	0.100 (3)	0.085 (2)	0.078 (2)	−0.007 (2)	0.035 (2)	−0.0181 (19)
C4	0.106 (3)	0.075 (2)	0.063 (2)	−0.003 (2)	0.0190 (18)	−0.0012 (16)
C5	0.079 (2)	0.0652 (18)	0.0626 (19)	0.0032 (15)	0.0113 (15)	0.0011 (15)
C6	0.0622 (16)	0.0500 (15)	0.0603 (17)	−0.0040 (12)	0.0143 (12)	−0.0008 (12)
C7	0.0490 (14)	0.0428 (13)	0.0662 (17)	−0.0034 (10)	0.0088 (12)	−0.0001 (12)
C8	0.0551 (15)	0.0489 (15)	0.0658 (17)	0.0006 (11)	0.0071 (12)	−0.0013 (12)
C9	0.0555 (16)	0.0664 (19)	0.076 (2)	0.0085 (13)	0.0058 (13)	0.0055 (15)
C10	0.0566 (15)	0.0512 (15)	0.0548 (15)	0.0017 (11)	0.0049 (11)	−0.0003 (12)
C11	0.0641 (18)	0.0492 (15)	0.081 (2)	0.0081 (13)	0.0120 (15)	0.0012 (14)
C12	0.0590 (17)	0.0669 (19)	0.085 (2)	0.0080 (14)	0.0157 (15)	0.0062 (16)
C13	0.0655 (18)	0.0618 (18)	0.071 (2)	0.0006 (14)	0.0020 (14)	0.0111 (14)
C14	0.083 (2)	0.0476 (16)	0.098 (2)	0.0051 (15)	0.0211 (18)	0.0097 (15)
C15	0.0688 (19)	0.0560 (18)	0.090 (2)	0.0118 (14)	0.0196 (16)	0.0005 (15)
C16	0.077 (2)	0.090 (3)	0.122 (3)	−0.0133 (19)	0.014 (2)	0.031 (2)
C17	0.0572 (16)	0.0496 (15)	0.0722 (18)	0.0004 (12)	0.0166 (13)	0.0017 (13)
C18	0.0643 (17)	0.0509 (15)	0.0748 (19)	0.0046 (12)	0.0159 (14)	−0.0026 (14)
C19	0.0553 (15)	0.0706 (18)	0.0576 (16)	0.0056 (13)	0.0120 (12)	0.0058 (14)
C20	0.0494 (16)	0.076 (2)	0.085 (2)	−0.0058 (14)	0.0087 (15)	0.0061 (16)
C21	0.0569 (17)	0.0540 (17)	0.104 (3)	−0.0059 (13)	0.0070 (16)	−0.0066 (16)
C22	0.064 (2)	0.095 (3)	0.078 (2)	0.0186 (19)	0.0220 (17)	0.002 (2)
O3A	0.078 (5)	0.159 (8)	0.101 (5)	0.047 (5)	0.037 (4)	0.034 (5)
O4A	0.068 (3)	0.064 (3)	0.122 (7)	0.012 (2)	0.031 (3)	−0.019 (3)
C23A	0.091 (5)	0.111 (9)	0.104 (7)	0.014 (6)	0.015 (5)	−0.006 (6)
C24A	0.093 (6)	0.091 (7)	0.153 (9)	−0.007 (5)	0.021 (6)	−0.010 (7)
O3B	0.058 (5)	0.167 (11)	0.177 (17)	0.009 (6)	0.039 (8)	0.054 (10)
O4B	0.129 (9)	0.149 (9)	0.141 (12)	0.072 (7)	0.062 (8)	0.008 (6)
C23B	0.107 (9)	0.134 (12)	0.144 (14)	0.056 (9)	0.068 (10)	0.002 (11)
C24B	0.093 (8)	0.059 (6)	0.229 (19)	0.025 (5)	0.050 (10)	−0.024 (8)

### Geometric parameters (Å, º)

**Table:**

S1—O1	1.428 (2)	C16—H16B	0.9600
S1—O2	1.438 (2)	C16—H16C	0.9600
S1—C8	1.771 (3)	C17—H17A	0.9700
S1—C10	1.755 (3)	C17—H17B	0.9700
N1—C1	1.366 (4)	C17—C18	1.518 (4)
N1—C9	1.306 (4)	C18—H18A	0.9700
N2—C7	1.419 (3)	C18—H18B	0.9700
N2—C17	1.469 (3)	C18—C19	1.523 (4)
N2—C21	1.476 (3)	C19—H19	0.9800
C1—C2	1.407 (4)	C19—C20	1.512 (4)
C1—C6	1.412 (4)	C19—C22	1.494 (3)
C2—H2	0.9300	C20—H20A	0.9700
C2—C3	1.367 (5)	C20—H20B	0.9700
C3—H3	0.9300	C20—C21	1.514 (4)
C3—C4	1.384 (5)	C21—H21A	0.9700
C4—H4	0.9300	C21—H21B	0.9700
C4—C5	1.364 (4)	C22—O3A	1.191 (5)
C5—H5	0.9300	C22—O4A	1.340 (4)
C5—C6	1.405 (4)	C22—O3B	1.194 (5)
C6—C7	1.431 (4)	C22—O4B	1.334 (5)
C7—C8	1.381 (4)	O4A—C23A	1.464 (5)
C8—C9	1.417 (4)	C23A—H23A	0.9700
C9—H9	0.9300	C23A—H23B	0.9700
C10—C11	1.381 (4)	C23A—C24A	1.502 (5)
C10—C15	1.383 (4)	C24A—H24A	0.9600
C11—H11	0.9300	C24A—H24B	0.9600
C11—C12	1.387 (4)	C24A—H24C	0.9600
C12—H12	0.9300	O4B—C23B	1.462 (5)
C12—C13	1.382 (4)	C23B—H23C	0.9700
C13—C14	1.383 (4)	C23B—H23D	0.9700
C13—C16	1.501 (4)	C23B—C24B	1.506 (5)
C14—H14	0.9300	C24B—H24D	0.9600
C14—C15	1.373 (4)	C24B—H24E	0.9600
C15—H15	0.9300	C24B—H24F	0.9600
C16—H16A	0.9600		
			
O1—S1—O2	117.38 (14)	N2—C17—H17B	109.7
O1—S1—C8	111.76 (13)	N2—C17—C18	109.8 (2)
O1—S1—C10	109.63 (13)	H17A—C17—H17B	108.2
O2—S1—C8	104.97 (13)	C18—C17—H17A	109.7
O2—S1—C10	106.93 (13)	C18—C17—H17B	109.7
C10—S1—C8	105.39 (13)	C17—C18—H18A	109.4
C9—N1—C1	116.9 (3)	C17—C18—H18B	109.4
C7—N2—C17	114.9 (2)	C17—C18—C19	111.2 (2)
C7—N2—C21	117.0 (2)	H18A—C18—H18B	108.0
C17—N2—C21	113.5 (2)	C19—C18—H18A	109.4
N1—C1—C2	116.7 (3)	C19—C18—H18B	109.4
N1—C1—C6	123.1 (3)	C18—C19—H19	107.7
C2—C1—C6	120.1 (3)	C20—C19—C18	109.5 (2)
C1—C2—H2	119.8	C20—C19—H19	107.7
C3—C2—C1	120.4 (3)	C22—C19—C18	112.9 (3)
C3—C2—H2	119.8	C22—C19—H19	107.7
C2—C3—H3	120.1	C22—C19—C20	111.1 (2)
C2—C3—C4	119.8 (3)	C19—C20—H20A	109.4
C4—C3—H3	120.1	C19—C20—H20B	109.4
C3—C4—H4	119.5	C19—C20—C21	111.3 (2)
C5—C4—C3	120.9 (3)	H20A—C20—H20B	108.0
C5—C4—H4	119.5	C21—C20—H20A	109.4
C4—C5—H5	119.3	C21—C20—H20B	109.4
C4—C5—C6	121.4 (3)	N2—C21—C20	109.8 (2)
C6—C5—H5	119.3	N2—C21—H21A	109.7
C1—C6—C7	118.5 (3)	N2—C21—H21B	109.7
C5—C6—C1	117.3 (3)	C20—C21—H21A	109.7
C5—C6—C7	124.2 (3)	C20—C21—H21B	109.7
N2—C7—C6	124.0 (2)	H21A—C21—H21B	108.2
C8—C7—N2	119.2 (2)	O3A—C22—C19	124.7 (8)
C8—C7—C6	116.8 (2)	O3A—C22—O4A	123.3 (9)
C7—C8—S1	123.0 (2)	O4A—C22—C19	111.9 (4)
C7—C8—C9	119.7 (3)	O3B—C22—C19	129.6 (13)
C9—C8—S1	117.3 (2)	O3B—C22—O4B	116.4 (14)
N1—C9—C8	124.6 (3)	O4B—C22—C19	113.9 (5)
N1—C9—H9	117.7	C22—O4A—C23A	123.1 (7)
C8—C9—H9	117.7	O4A—C23A—H23A	110.5
C11—C10—S1	120.9 (2)	O4A—C23A—H23B	110.5
C11—C10—C15	120.0 (3)	O4A—C23A—C24A	106.0 (8)
C15—C10—S1	118.9 (2)	H23A—C23A—H23B	108.7
C10—C11—H11	120.3	C24A—C23A—H23A	110.5
C10—C11—C12	119.5 (3)	C24A—C23A—H23B	110.5
C12—C11—H11	120.3	C23A—C24A—H24A	109.5
C11—C12—H12	119.4	C23A—C24A—H24B	109.5
C13—C12—C11	121.3 (3)	C23A—C24A—H24C	109.5
C13—C12—H12	119.4	H24A—C24A—H24B	109.5
C12—C13—C14	118.0 (3)	H24A—C24A—H24C	109.5
C12—C13—C16	120.7 (3)	H24B—C24A—H24C	109.5
C14—C13—C16	121.4 (3)	C22—O4B—C23B	107.5 (9)
C13—C14—H14	119.1	O4B—C23B—H23C	111.1
C15—C14—C13	121.8 (3)	O4B—C23B—H23D	111.1
C15—C14—H14	119.1	O4B—C23B—C24B	103.2 (8)
C10—C15—H15	120.3	H23C—C23B—H23D	109.1
C14—C15—C10	119.4 (3)	C24B—C23B—H23C	111.1
C14—C15—H15	120.3	C24B—C23B—H23D	111.1
C13—C16—H16A	109.5	C23B—C24B—H24D	109.5
C13—C16—H16B	109.5	C23B—C24B—H24E	109.5
C13—C16—H16C	109.5	C23B—C24B—H24F	109.5
H16A—C16—H16B	109.5	H24D—C24B—H24E	109.5
H16A—C16—H16C	109.5	H24D—C24B—H24F	109.5
H16B—C16—H16C	109.5	H24E—C24B—H24F	109.5
N2—C17—H17A	109.7		
			
S1—C8—C9—N1	−175.8 (2)	C9—N1—C1—C2	−179.0 (3)
S1—C10—C11—C12	175.7 (2)	C9—N1—C1—C6	0.5 (4)
S1—C10—C15—C14	−176.9 (3)	C10—S1—C8—C7	−71.5 (3)
O1—S1—C8—C7	47.6 (3)	C10—S1—C8—C9	106.4 (2)
O1—S1—C8—C9	−134.6 (2)	C10—C11—C12—C13	0.5 (5)
O1—S1—C10—C11	4.5 (3)	C11—C10—C15—C14	−1.9 (5)
O1—S1—C10—C15	179.5 (2)	C11—C12—C13—C14	−0.6 (5)
O2—S1—C8—C7	175.8 (2)	C11—C12—C13—C16	179.5 (3)
O2—S1—C8—C9	−6.4 (3)	C12—C13—C14—C15	−0.5 (5)
O2—S1—C10—C11	−123.7 (2)	C13—C14—C15—C10	1.7 (5)
O2—S1—C10—C15	51.3 (3)	C15—C10—C11—C12	0.8 (4)
N1—C1—C2—C3	176.8 (3)	C16—C13—C14—C15	179.4 (3)
N1—C1—C6—C5	−175.5 (3)	C17—N2—C7—C6	83.1 (3)
N1—C1—C6—C7	4.2 (4)	C17—N2—C7—C8	−98.2 (3)
N2—C7—C8—S1	1.7 (4)	C17—N2—C21—C20	57.5 (3)
N2—C7—C8—C9	−176.1 (2)	C17—C18—C19—C20	−55.7 (3)
N2—C17—C18—C19	55.9 (3)	C17—C18—C19—C22	179.8 (2)
C1—N1—C9—C8	−3.7 (5)	C18—C19—C20—C21	55.9 (3)
C1—C2—C3—C4	−1.0 (5)	C18—C19—C22—O3A	117.3 (10)
C1—C6—C7—N2	173.2 (2)	C18—C19—C22—O4A	−61.1 (6)
C1—C6—C7—C8	−5.5 (4)	C18—C19—C22—O3B	143.1 (14)
C2—C1—C6—C5	3.9 (4)	C18—C19—C22—O4B	−32.5 (9)
C2—C1—C6—C7	−176.4 (3)	C19—C20—C21—N2	−56.2 (4)
C2—C3—C4—C5	3.3 (5)	C19—C22—O4A—C23A	−178.2 (7)
C3—C4—C5—C6	−1.9 (5)	C19—C22—O4B—C23B	−177.9 (9)
C4—C5—C6—C1	−1.7 (5)	C20—C19—C22—O3A	−6.2 (11)
C4—C5—C6—C7	178.7 (3)	C20—C19—C22—O4A	175.4 (5)
C5—C6—C7—N2	−7.1 (4)	C20—C19—C22—O3B	19.6 (15)
C5—C6—C7—C8	174.1 (3)	C20—C19—C22—O4B	−156.0 (9)
C6—C1—C2—C3	−2.6 (5)	C21—N2—C7—C6	−53.7 (4)
C6—C7—C8—S1	−179.56 (19)	C21—N2—C7—C8	124.9 (3)
C6—C7—C8—C9	2.7 (4)	C21—N2—C17—C18	−57.4 (3)
C7—N2—C17—C18	164.2 (2)	C22—C19—C20—C21	−178.7 (3)
C7—N2—C21—C20	−165.0 (2)	C22—O4A—C23A—C24A	−98.1 (14)
C7—C8—C9—N1	2.1 (5)	C22—O4B—C23B—C24B	150 (2)
C8—S1—C10—C11	124.9 (2)	O3A—C22—O4A—C23A	3.4 (14)
C8—S1—C10—C15	−60.1 (3)	O3B—C22—O4B—C23B	5.9 (17)

### Hydrogen-bond geometry (Å, º)

**Table:**

*D*—H···*A*	*D*—H	H···*A*	*D*···*A*	*D*—H···*A*
C4—H4···O3*A*^i^	0.93	2.52	3.421 (16)	163
C5—H5···O2^ii^	0.93	2.58	3.415 (4)	149

Symmetry codes: (i) *x*−1/2, −*y*+3/2, *z*+1/2; (ii) *x*+1/2, −*y*+3/2, *z*+1/2.
