# Supplementary material for: Synthesis, X-ray diffraction study, analysis of inter­molecular inter­actions and mol­ecular docking of ethyl 1-(3-tosyl­quinolin-4-yl)piperidine-4-carboxyl­ate
Source: Acta Crystallogr E Crystallogr Commun. 2022 Aug 9;78(Pt 9):890–6. doi: 10.1107/S2056989022007691 (PMC9443802; doi:10.1107/S2056989022007691)
Supplement: Supplementary file 3 [file e-78-00890-sup3.pdf]

The parameters used in molecular docking:

· The maximum RMS tolerance for the conformational cluster analysis – 2 Å.

· The free energy coefficient for torsional degrees of freedom – 0.2983.

· The cluster tolerance – 2 Å.

· The external grid energy – 1000.

· The maximum initial energy – 0; the maximum number of retries – 10 000.

· The number of individuals in the population – 150.

· The maximum number of energy evaluations – 2500000.

· The maximum number of generations – 27 000.

· The number of top individuals to survive to the next generation – 1.

· The rate of gene mutation – 0.02; the rate of crossover – 0.8.

· The crossover mode – arithmetic; the α-parameter of Gauss distribution – 0.

· The β-parameter of Gauss distribution – 1.
